# The effects of subcutaneous Tirzepatide on obesity and overweight: a systematic review and meta‐regression analysis of randomized controlled trials

**DOI:** 10.3389/fendo.2023.1230206

**Published:** 2023-08-09

**Authors:** Pejman Rohani, Nasser Malekpour Alamdari, Seyedeh Elaheh Bagheri, Azita Hekmatdoost, Mohammad Hassan Sohouli

**Affiliations:** ^1^ Pediatric Gastroenterology and Hepatology Research Center, Pediatrics Centre of Excellence, Children’s Medical Center, Tehran University of Medical Sciences, Tehran, Iran; ^2^ Department of General Surgery, School of Medicine, Shahid Modarres Hospital, Shahid Beheshti University of Medical Sciences, Tehran, Iran; ^3^ Tehran University of Medical Sciences, Tehran, Iran; ^4^ Department of Clinical Nutrition and Dietetics, Faculty of Nutrition and Food Technology, Shahid Beheshti University of Medical Sciences, Tehran, Iran; ^5^ Student Research Commitee, Department of Clinical Nutrition and Dietetics, Faculty of Nutrition and Food Technology, Shahid Beheshti University of Medical Sciences, Tehran, Iran

**Keywords:** tirzepatide, obesity, overweight, glycemic control, weight loss

## Abstract

**Background:**

Despite the fact that obesity and overweight are serious major health problems worldwide, fighting against them is also considered a challenging issue. Several interventional studies have evaluated the potential weight-reduction effect of Tirzepatide. In order to obtain a better viewpoint from them, this study aimed to comprehensively investigate the effects of subcutaneous Tirzepatide on obesity and overweight.

**Methods:**

Scopus, PubMed/Medline, Web of Science, Cochrane, and Embase databases were searched using standard keywords to identify all controlled trials investigating the weight loss effects of Tirzepatide. Pooled weighted mean difference and 95% confidence intervals were achieved by random-effects model analysis for the best estimation of outcomes. The statistical heterogeneity and publication bias were determined using the Cochran’s *Q* test and I^2^ statistics and using the funnel plot and Egger’s test, respectively.

**Results:**

Twenty three treatments arm with 7062 participants’ were included in this systematic review and meta‐regression analysis. The pooled findings showed that Tirzepatide vs placebo significantly reduced body weight (weighted mean difference (WMD): -11.34 kg, 95% confidence interval (CI): -12.79 to -9.88, P< 0.001), body mass index (BMI) (WMD: -3.11 kg/m2, 95% CI: -4.36 to -1.86, P< 0.001), and waist circumference (WC) (WMD: -7.24 cm, 95% CI -10.12 to -4.36, P< 0.001). These reductions were even greater, especially with higher doses and duration of Tirzepatide.

**Conclusions:**

Tirzepatide medication had significant effects on weight management with the reduction of body weight, BMI, and WC. Administration of Tirzepatide can be considered a therapeutic strategy for overweight or obese people.

## Introduction

Obesity and overweight are two of the main health challenges worldwide that affect over a third of the population around the world (1). They are the leading causes of death, contributing to at least 2.8 million deaths and 35.8 million global disability-adjusted life years (DALYs), according to the most recent reports from the World Health Organization (WHO) (2). A wide range of metabolic alterations and clinical anomalies are present in people with persistent excessive weight gain or obesity. Additionally, untreated overweight or obese individuals are linked to a number of complications, such as chronic diseases like diabetes mellitus, hypertension, cardiovascular disease, and stroke (3–6). Moreover, recent evidence has shown that these population groups are not only at a higher risk of contracting non-communicable diseases but also at a higher risk of poorer outcomes from communicable diseases, such as increased rate of hospitalization and mortality from viral infections like severe acute respiratory syndrome coronavirus-2 (SARS-CoV-2) as compared with the general population (3, 4).

In general, the causes of obesity are multifaceted and difficult to pinpoint (1, 7), although they can include environmental and lifestyle variables including physical activity and food as well as hereditary factors (6, 8). So, it is expected that lifestyle change strategies should be done to prevent and combat obesity. However, the fight against obesity has been one of the greatest challenges. Indeed, it is found that even with lifestyle changes, the success rate in weight loss is not always satisfactory (6, 9). Therefore, given the high importance of obesity as the most common risk factor for developing the disease (1, 5, 6), clinical management primarily emphasizes weight loss, which can be achieved through medication.

According to several new clinical guidelines, the role of anti-obesity medications for obese or overweight people who have weight-related complications is highlighted as a recommended treatment for obesity (10). Glucagon-like peptide-1 (GLP-1) and gastric inhibitory polypeptide (GIP), as two of the main incretin peptide hormones, are responsible for glucose homeostasis and enhance glucose-stimulated insulin secretion after nutrient ingest (11). The distal ileum and colon’s L cells generate the 30 amino acid peptide GLP-1, whereas the duodenum and jejunum’s cells create the 42 amino acid peptide GIP (12). It is probable that the successful application of incretin peptide hormones has the potential to reduce weight and consider an obesity treatment (13, 14). Interestingly, more recent evidence has shown that the combination of both GIP and GLP-1 through multiple metabolic mechanisms synergistically affect each other. Therefore, it is conceivable that compared to administering each hormone separately, this may result in significantly increased insulin and glucagonostatic responses, which may have a greater impact on the effectiveness of weight loss (15, 16).

The synthetic peptide tirzepatide (LY3298176) as a dual agonist of GIP and GLP-1, which is used as a subcutaneous injection once a week, has recently attracted the attention of scientists as a peptide containing 39 amino acids (17, 18) that raises insulin secretion, lowers glucagon secretion, delays gastric emptying, lowers dietary intake, and ultimately lowers body weight (19–21). Reduced hepatic glucose synthesis and plasma glucose levels seem to be linked to GIP and GLP-1 agonist’s capacity to inhibit glucagon release. This medicine is an FDA-approved treatment for type 2 diabetic mellitus (T2DM) based on scientific data (15, 22, 23). Moreover, in a phase 3 clinical trial study on 2400 people living with obesity and overweight, it showed the effectiveness of Tirzepatide on anthropometry parameters (10). Despite some of the gastrointestinal adverse events of Tirzepatide including nausea, vomiting, and diarrhea (6, 24, 25), it has superiority to other similar agents and is considered a promising anti-obesity therapeutic drug due to its multiple pharmacological targets on nutrient-stimulated hormone receptor agonists (6, 26). However, the appropriate dose and duration of Tirzepatide therapy which is effective in weight loss is an issue that needs more comprehensive studies (27). The present systematic review and meta-regression analysis, based on clinical trials, aimed to investigate the effects of Tirzepatide on weight loss.

## Methods

### Search strategy

The Preferred Reporting Items for Systematic Review and Meta-analysis (PRISMA) criteria were followed for conducting this study (28). Without regard to language or time restrictions, a thorough search was carried out in the PubMed/MEDLINE, Web of Science, SCOPUS, and Embase databases from the December 2018 to August 2022. Additionally, similar papers and gray literature were considered in the search. Medical subject headings (MeSH) and Emtree (Embase subject headings) were selected to search the online databases, as follow: (Tirzepatide OR ly3298176) AND (“weight” OR “Waist Circumference” OR “Body Mass Index”) AND (“Clinical Trials as Topic” OR “Cross-Over Studies” OR “Double-Blind Method” OR “Single-Blind Method” OR “Random Allocation” OR “Clinical Trial”) (The search strategy was added to the appendix as an example in the PubMed search database). In order to discover potentially overlooked eligible trials, the reference lists of the papers found and associated review studies were also manually examined.

### Eligibility criteria

Using titles, abstracts, or the complete texts of the research, two writers separately removed duplicate articles before finding and reviewing relevant publications. Discrepancy rate between reviewers was less than 4%, which was resolved by the third reviewer. In the end, the papers were separated based on the following standards: 1) Randomized clinical trials studies; 2) Tirzepatide was given as an intervention to adults; 3) The existence of a control group in the form of placebo or insulin, and 4) Baseline and post in both group (intervention and control) weight, WC, and BMI were recorded. If a study revealed outcomes at more than one follow-up period, the most recent or most extensive follow-up time was taken into account. Studies with duplicated data, studies with unclear information, studies in which Tirzepatide was an intervention in conjunction with other widely used medications, non-randomized trial designs, animal studies, studies without a control group, and reviews or meta-analysis studies were also omitted. Also, if Tirzepatide is compared with other common drugs or drugs with similar performance, the desired study will be removed due to lack of sufficient effect of Tirzepatide.

### Data extraction

Two authors independently reviewed the eligible studies. Name of first author, study site, year of publication, RCT design (crossover or parallel), sample size (intervention and control groups), participant characteristics (gender, BMI, age, and health status), type of outcomes, length of intervention, dosage of intervention, and means and standard deviations (S.D.s) of intended outcomes at baseline, post-intervention, and/or changes between baseline and post-intervention were all extracted.

### Quality assessment

The details of the study quality assessment are presented in **Table 1**. The quality of the included RCTs was methodologically assessed using the Cochrane risk-of-bias test for randomized trials (RoB 2), version 2 (37). Two authors independently rated each study as having a low, high, or unclear risk of bias based on the following potential sources of bias: blinding of outcome assessment, allocation concealment, participant and staff blinding, random sequence generation, incomplete outcome data, selective reporting, and other bias. Any disagreements were discussed with a third author to find a solution. In order to assess the quality of the current analysis study, the NutriGrade (Grading of Recommendations Assessment, Development, and Evaluation) grading system was also utilized (38). A reliable 10-point assessment system that assesses elements affecting study quality is the NutriGrade checklist. This scale has seven components: (1) risk of bias, (2) precision, (3) heterogeneity, (4) directness, (5) publishing bias, (6) funding bias, and (7) study design.

**Table 1:** Risk of bias assessment according to the Cochrane collaboration's risk of bias assessment tool.

Study, Year (reference)	Random sequence generation	Allocation concealment	Blinding of participant and its personnel	Blinding of outcome assessment	Incomplete outcome data	Selective reporting	Overall assessment of risk of bias
***Del Prato et al.*** (29)	Low	Low	Low	Low	Unclear	Low	Low
***Heise et al.*** (30)	Low		Low	Low	Unclear	Low	Unclear
***Dahl et al.*** (31)	Low	Low	Low	Low	Unclear	Low	Low
***Jastreboff et al. (*** 32)	Low	Low	Low	Low	Unclear	Low	Low
***Rosenstock et al. (*** 33)	Low	Unclear	Low	Low	Unclear	Low	Unclear
***Gastaldelli et al.*** (34)	Low	Low	Low	Low	Unclear	Low	Low
***Ludvik et al.*** (35)	Low	Low	Low	Low	Unclear	Low	Low
***Frias et al.*** (36)	Low	Low	Low	Low	Unclear	Low	Low

### Data synthesis and statistical analysis

STATA version 12.0 software was used to analyze the data. Different data formats were converted to the mean and standard deviations (S.D.s) using established formula (39, 40). For instance, we estimated the change using the formula below in the absence of standard deviations: Square root [(S.D. baseline ^2^ + SD final ^2^) - (2R S.D. baseline 2 S.D. final)] is the definition of S.D. changes. The following formula is used to convert the standard error of the mean (SEM) to standard deviation: S.D. is equal to SEM × √n, where n is the total number of participants in each group. The meta-analysis of study findings was conducted using the random-effects model. The general inverse variance approach was used to weight the research. Multiple evaluations within a single research group were handled by using the values from the longest time point for the analysis. The status of study heterogeneity was assessed using Q Statistics and I-squared (I^2^). With I^2^ values ranging from 0% to 25, 26% to 50%, 5% to 75%, and 76% to 100%, respectively, insignificant, low, moderate, and high heterogeneity were detected (41). Meta-regression investigates whether particular covariates (potential effect modifiers) explain any of the heterogeneity of treatment effects between studies. Thus, meta-regression between subcutaneous Tirzepatide and absolute mean differences in body weight based on dosage, baseline of mean age and BMI, and duration of intervention was performed using random effect model. A pre-defined subgroup analysis based on the dosage of the intervention was carried out to find potential sources of heterogeneity. To determine the contribution of each study to the total mean difference, a sensitivity analysis was used. We used the official Egger’s test to determine whether there was publication bias (42).

## Results

**Figure 1:**
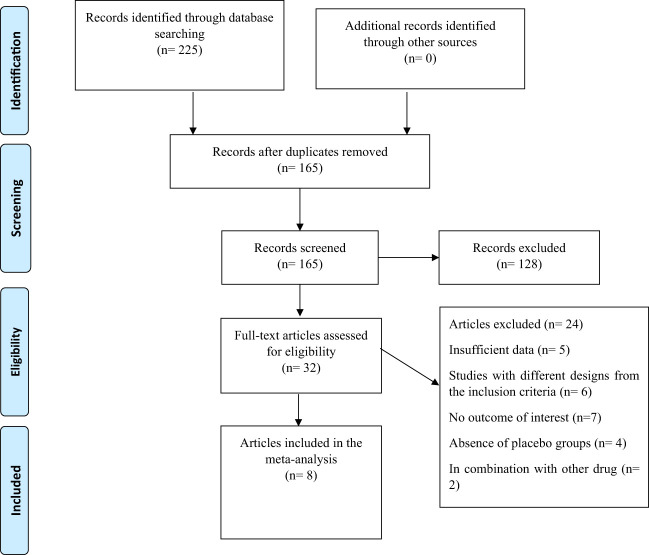
Flow chart of the study, including identification, screening, eligibility, and the final sample included.

A flowchart of the study selection procedure with exclusion criteria is shown in **Figure 1**. The aforementioned electronic databases produced 225 publications, according to this number. There were 165 papers overall after duplicate research were eliminated. Following a review of the titles and abstracts of the research, 128 publications were eliminated because they did not match the criteria for inclusion. During the secondary screening, 32 articles were located using full-text. 24 of the studies were eliminated for the aforementioned reasons. Finally, 8 papers (29–36) with 23 treatments arm were included in the quantitative meta-analysis since they matched the qualifying requirements.

### Study characteristics

The features of the pooled articles are shown in **Table 2**. Our surveys reveal that one study have been carried out in Germany and other studies were conducted in a multi-center or multi-country manner. Every study was released between 2018 and 2022. The all of the research utilized a parallel design and follow up intervention ranged from 26 to 72 weeks. The mean age and percentage of male participants ranged from 45 to 63.4 years and 31-62%, respectively, at the baseline. Six studies conducted on individuals with type 2 diabetes and two studies on participants living with obesity. Also, intervention doses ranged from 1 to 10 mg subcutaneously.

**Table 2:** Characteristics of eligible studies.

Author (year)	Country	Study phase	Population	Mean Age year	Sex (Male %)	Sample Size Study	Follow up of intervention (Weeks)	Dose (mg) of intervention	Baseline of BMI (kg/m^2^)	Outcomes
***Del Prato et al. 2021*** (29)	*Multi-center (Argentina, Australia, Brazil, Canada, Greece, Israel, Mexico, Poland, Romania, Russia, Slovakia, Spain, Taiwan, and the USA)*	*3*	*Type 2 diabetes*	*63.4*	*62*	*1985*	*52*	*once-weekly subcutaneous 5, 10, and 15 mg*	*32.6*	*Weight*
***Heise et al. 2022*** (30)	*Germany*	*1*	*Type 2 diabetes*	*61.1*	*31*	*73*	*28*	*once-weekly subcutaneous 15 mg*	*31.2*	*Weight*
***Dahl et al. 2020*** (31)	*Multi-center (USA, Japan, Czech Republic, Germany, Poland, Puerto Rico, Slovakia, and Spain)*	*3*	*Type 2 diabetes*	*61*	*56*	*475*	*40*	*once-weekly subcutaneous 5, 10, and 15 mg*	*33.5*	*Weight*
***Jastreboff et al. 2022*** (32)	*Multi-center (Countries not reported(*	*3*	*Obesity*	*45*	*32.6*	*2539*	*72*	*once-weekly subcutaneous 5, 10, and 15 mg*	*37.9*	*Weight*
***Rosenstock et al.2021*** (33)	*Multi-center (India, Japan, Mexico, and the USA)*	*3*	*Type 2 diabetes*	*54.2*	*52.6*	*478*	*40*	*once-weekly subcutaneous 5, 10, and 15 mg*	*31.8*	*Weight*
***Gastaldelli et al.2022*** (34)	*Multi-center (Argentina, Austria, Greece, Hungary, Italy, Romania, Spain, and the USA)*	*3*	*Type 2 diabetes*	*56.2*	*74*	*296*	*52*	*once-weekly subcutaneous 5, 10, and 15 mg*	*33.5*	*Weight, BMI, WC*
***Ludvik et al. 2021*** (35)	*Multi-center (Argentina, Austria, Greece, Hungary, Italy, Poland, Puerto Rico, Romania, South Korea, Spain, Taiwan, Ukraine, and the USA)*	*3*	*Type 2 diabetes*	*57.3*	*55*	*954*	*52*	*once-weekly subcutaneous 5, 10, and 15 mg*	*33.5*	*Weight*
***Frias et al. 2018*** (36)	*Multi-center (Poland, Puerto Rico, Slovakia, and USA)*	*2*	*Type 2 diabetes*	*56.9*	*54.7*	*262*	*26*	*once-weekly subcutaneous 1, 5, 10, and 15 mg*	*32.6*	*Weight, BMI, WC*

BMI, body mass index; WC, waist circumferences.

The findings of the evaluation of the eligible studies’ quality are shown in **Table 1**. Additionally, a score of 9.6 (very good quality) was determined after the NutriGrade score system was used to assess the quality of the current meta-analysis.

### Meta-analysis results

**Figure 2:**
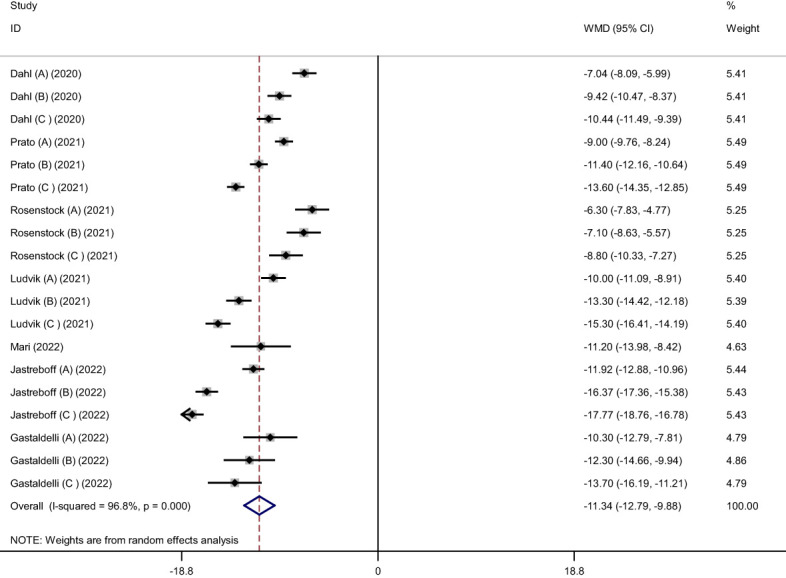
Forest plot of randomized controlled trials investigating the effects of Tirzepatide on weight (kg).

**Figure 3:**
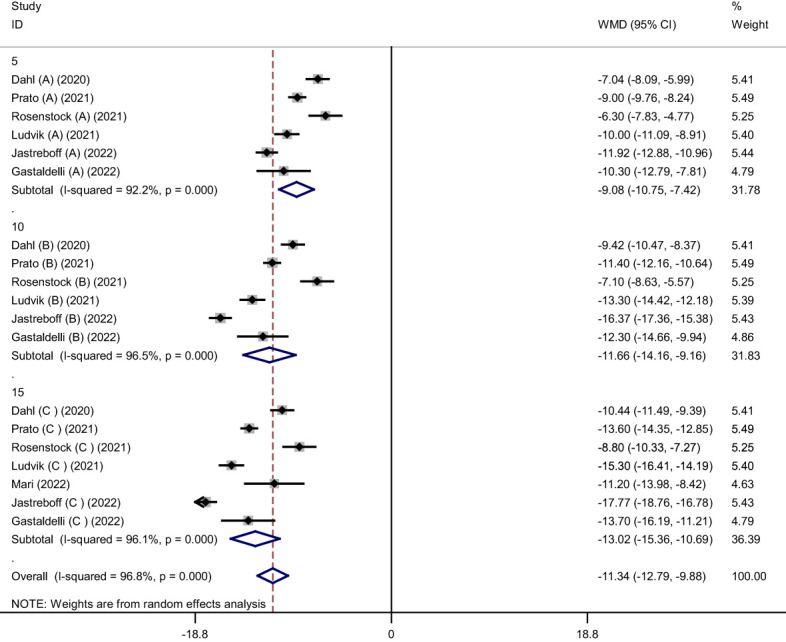
Forest plot of randomized controlled trials investigating the effects of Tirzepatide on weight based on dose of intervention (mg).

**Figure 4:**
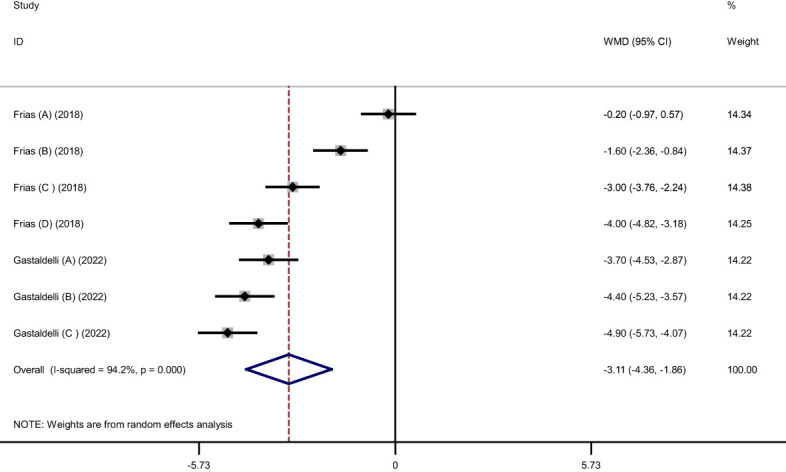
Forest plot of randomized controlled trials investigating the effects of Tirzepatide on body mass index (BMI) (kg/m^2^).

**Figure 5:**
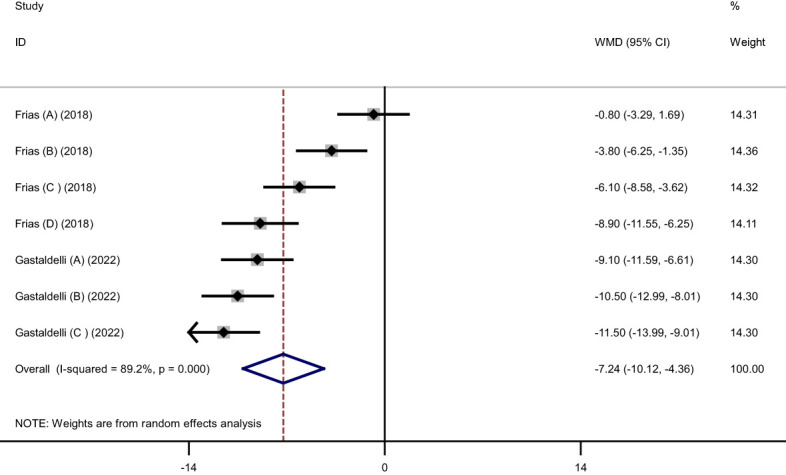
Forest plot of randomized controlled trials investigating the effects of Tirzepatide on waist circumference (WC) (cm).

Pooled findings from the random-effects model indicated that body weight (weighted mean difference (WMD): -11.34 kg, 95% confidence interval (CI): -12.79 to -9.88, P< 0.001), body mass index (BMI) (WMD: -3.11 kg/m^2^, 95% CI: -4.36 to -1.86, P< 0.001), and waist circumference (WC) (WMD: -7.24 cm, 95% CI -10.12 to -4.36, P< 0.001) were significantly reduced after subcutaneous Tirzepatide compared to control group. Also, subgroup results showed that changes in weight loss following subcutaneous Tirzepatide at a dose of 15 mg (WMD: -13.02 kg, 95% CI: -15.36 to -10.69, I^2 =^ 96.1%) were higher compared to other doses (10 mg (WMD: -11.66 kg, 95% CI: -14.16 to -9.16, I^2 =^ 96.5%), 5 mg (WMD: -9.08 kg, 95% CI: -10.75 to -7.42, I^2 =^ 92.2%)). Furthermore, significant heterogeneity was found among the studies for weight (Cochran *Q* test, P< 0.001, I^2 =^ 96.8%), BMI (Cochran *Q* test, P< 0.001, I^2 =^ 94.2%), and WC (Cochran *Q* test, P< 0.001, I^2 =^ 89.2%; **Figures 2**–**5**).

### Meta-regression

Meta-regression between subcutaneous Tirzepatide and absolute mean differences in body weight based on dosage, baseline of mean age and BMI, and duration of intervention was performed. There was a significant relationship between duration of intervention (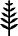), baseline of mean BMI (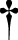) and dose of intervention (*) with changes in body weight (coefficient (Coef)= -0.1904459, P= 0.001 for 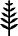; Coef= -0.990465, P= 0.004 for 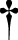; Coef= -0.3924448, P= 0.023 for *) (**Supplementary Figure 1**).

### Sensitivity analysis

We gradually removed each trial from the analysis to determine the impact of each article on the pooled effect size for the levels of weight, BMI, and WC. The robustness of the findings was demonstrated by the leave-one-out sensitivity analysis (**Supplementary Figure 2**).

### Publication bias

Inspecting the funnel visually to determine publication bias, the Egger’s tests for weight (P= 0.326), BMI (P= 0.056), and WC (P= 0.293) revealed no indication of bias (**Supplementary Figure 3**).

## Discussion

As far as we are aware, this is the first research to thoroughly assess and analyze the results of intervention trials on the impacts of subcutaneous Tirzepatide on weight reduction. According to the study’s findings, subcutaneous Tirzepatide significantly reduced weight, BMI, and waist circumference in the intervention group compared to the control group. Additionally, analyses revealed that parameters were particularly impacted by the dosage and duration of the Tirzepatide drug.

Tirzepatide has been approved by FDA in May 2022. Since it has revealed significant improvement in glycemic control and weight loss compared to other alternatives, currently it can now be recommended off-label for the treatment of obesity and implemented as a second-line T2DM medication, maximizing similar advantages that are reported with established GLP-1 medications (43, 44). On this matter, The SURPASS clinical trial program’s goal in this area was to examine the effectiveness and safety of tirzepatide in T2DM patients. In comparison to other well-known GLP-1 agonists used for weight loss management, such as Semaglutide, Tirzepatide has a strong effect on reducing glucose and body weight, according to the SURPASS Pre-clinical studies, 4-week-long phase 1 and 26-week-long phase 2 clinical trials. Tirzepatide showed dose-dependent benefits on the decrease of HbA1c and weight greater than 2.4% and 11.3 kg, respectively, in phase 1 and phase 2 studies (45). Moreover, a study conducted by Heise et al. reported a significant reduction in weight after intervention with Tirzepatide versus placebo or Semaglutide after 28 weeks in T2DM adults aged 20 to 74 years. Also due to the potent glucose-lowering effects of Tirzepatide, a significant reduction in glucagon secretion, as well as improvements in insulin sensitivity and β-cell function were observed (30). Consistent with the previous studies, Frias et al. investigated the efficacy and safety of Tirzepatide after 26 weeks among T2DM patients aged 18–75 years. They found that Tirzepatide caused greater reductions in HbA1, BMI, and WC compared to placebo. Also, greater dose of Tirzepatide had a higher efficacy on HbA1 control and weight loss (36).

Del Prato et al. assessed the efficacy and safety of Tirzepatide after 52 weeks on T2DM adults with high cardiovascular risk aged equal to or more than 18 years. They showed that tirzepatide improved weight at the end of the intervention compared to baseline. Also, the amount of weight loss was higher in the higher dose of tirzepatide. Regarding the cardiovascular perspective, both the risk and numbers of cardiovascular events were significantly lower among treatment groups (29). Another study conducted by Wilson et al. compared the effects of subcutaneous Tirzepatide versus placebo added to titrated insulin Glargine after 40 weeks on T2DM patients with mean age of 60 years. There was a significant reduction in weight after the intervention, especially with a greater dose of Tirzepatide. Also, changes in weight were higher in Tirzepatide receivers compared to placebo receivers (46). Singh et al. compared the effects of Tirzepatide versus insulin glargine after 52 weeks on T2DM patients with high cardiovascular risk aged equal to or more than 18 years. The improvement in weight changes between treatment groups suggests that Tirzepatide can be considered a good option (47).

Furthermore, a study conducted by Jastreboff et al. evaluated the efficacy and safety of Tirzepatide after 72 weeks on participants living with obese or overweight without diabetes aged equal to or more than 18 years. The results showed that changes in weight and WC following Tirzepatide at a greater dose of 15 mg were higher compared to its lower doses and placebo (32). On the other hand, Rosenstock et al. found that the dose-dependent effects of intervention with Tirzepatide on weight loss were higher after 40 weeks compared to baseline and placebo in T2DM patients aged 54·1 years. Also, due to the robust improvements in controlling glycemia and body weight, without increased risk of hypoglycemia, authors proposed the use of Tirzepatide as a potential monotherapy for T2DM treatment (33).

Each drug may cause unwanted side effects in addition to its required effects. The studies list stomach pain as one of the side effects that are most frequently reported, along with other less frequent side effects like gaseous stomach pain, heartburn, recurrent fever, skin itching, rash, redness, fullness in the stomach, swelling of the face, throat, and tongue, vomiting, and yellow eyes or skin.

In a meta-analysis study on adults living with obesity and no diabetes, Liraglutide as a GLP-1 receptor agonist reduced body weight (WMD −3.35 kg; 95%CI −4.65 to −2.05), and BMI (WMD −1.45 kg/m2; 95%CI −1.98 to −0.91) in comparison to placebo (48). In another meta-analysis in 2021, Liraglutide caused a weight loss of -4.19 kg (95%CI, -4.84 to -3.55) compared to the control group (49). In the case of another GLP-1 agonist, a recent meta-analysis (including 4 clinical trial studies) was conducted to investigate the effectiveness of subcutaneous Semaglutide compared to placebo for weight loss in adults with obesity and without diabetes, and it was shown that this drug causes weight loss by − 11.62 kg (95% CI: −13.03 to −10.21) (50). These findings were also reported in another meta-analysis for weight and body mass index (decrease by -4.48 kg/m2) (51). However, in another meta-analysis study in diabetic subjects (52), Semaglutide reduced weight by WMD: -2.73 kg and -4.09 kg, for 0.5 mg and 1 mg, respectively. Overall, the efficacy of Tirzepatide appears to be much greater than Liraglutide, but comparing it with Semaglutide requires more study to confirm the findings. Anti-diabetic medications were divided into three categories depending on how well they helped people lose weight. We defined a weak impact as a weight loss of less than 3.2% of one’s starting weight, a moderate effect as a weight loss between 3.2% and 5%, and a strong effect as a weight loss of more than 5%. The majority of writers discovered that metformin caused a small decrease in body weight (less than 3.2% of initial weight in all trials) (53). Acarbose reduces intestinal glucose absorption, which results in a decrease in daily calorie intake, however the effect on body weight is minimal (54). SGLT-2 I causes a statistically significant decrease in body weight and, in a dose-dependent manner, increases urine glucose excretion (55). The majority of weight loss caused by SGLT-2i is fat loss rather than lean mass loss, with visceral fat loss being somewhat larger than subcutaneous fat loss in T2D patients (55). Except for empagliflozin, which causes a mild weight reduction, SGLT-2 I has a moderate effect on weight loss (56). The anti-diabetic drug class that has demonstrated the highest efficacy in terms of weight loss is GLP1-RA (9). Exenatide’s and dulaglutide’s weight-related effects were low and minor, respectively, but liraglutide—the only GLP1-RA authorized for the treatment of obesity—tirzepatide, and semaglutide had a significant weight-related effect (57). A potential treatment option for weight loss is the dual GIP and GLP-1 receptor agonist, LY3298176, which outperforms dulaglutide in terms of effectiveness. Intriguing results with regard to lowering waist circumference have also been seen with this medication (36).

The present study has several major strengths. First, this is the first systematic review and meta-regression analysis investigating the effects of subcutaneous Tirzepatide on weight loss. Second, the causal inference of our results is strong due to the design of meta-regression analysis based on eligible clinical trials. Third, we considered the Cochrane Bias Methods to minimize systematic errors and achieve reliable estimates of effects. Forth, the caliber of the included papers was fairly high and the major findings held up well after sensitivity analyses and Egger’s test. Finally, the results of our study may contribute to determining a medication that specialists should take in mind and consider at least for patients who are highly at risk for continued abnormal weight gain or obesity progression.

However, our study had some limitations that jeopardized the extraction of robust conclusions. Clinically and statistically significant heterogeneities were found. These may be explained by the differences in the intervention-specific factors (e.g., type, dose, administration route, and duration of drugs) and weight-specific factors (e.g., age, sex, physiology, genetics, familial history, race/ethnicity, physical activity, socioeconomic status, dietary intakes, and drug, tobacco, or alcohol consumption) (58). Nonetheless, we attempted to identify some possible sources of heterogeneity in data by performing a subgroup analysis.

Taken together, Tirzepatide is a novel medication approved for treating T2DM with the extra benefit of weight loss.

## Conclusions

In general, the present systematic review and meta‐regression analysis demonstrated that subcutaneous Tirzepatide may be able to significantly improve body weight, BMI, and WC in the intervention group compared to the control. The beneficial effect seemed greatest in those trials with higher doses and duration of Tirzepatide. Multidimensional weight loss management, such as combination with other related medications and lifestyle interventions might optimize the therapeutic effect of Tirzepatide for overweight or obese people. Further homogeneous and well-powered clinical trials on the appropriate dose and duration of Tirzepatide medication for different ranges of overweight persons are required to confirm our findings and increase our understanding of the effects of subcutaneous Tirzepatide on weight loss.

## Data availability statement

The datasets presented in this article are not readily available due to privacy/ethical restrictions. Requests to access the datasets should be directed to mohammadhassansohouli@gmail.com.

## Author contributions

PR, AH, and MS contributed in conception, design, and statistical analysis. MS, AH, NM, SB, and PR contributed in data collection and manuscript drafting. MS and AH supervised the study. All authors approved the final version of the manuscript.
