# Supplementary material for: The effects of subcutaneous Tirzepatide on obesity and overweight: a systematic review and meta‐regression analysis of randomized controlled trials
Source: Front Endocrinol (Lausanne). 2023 Aug 9;14:1230206. doi: 10.3389/fendo.2023.1230206 (PMC10446893; doi:10.3389/fendo.2023.1230206)
Supplement: Supplementary file 1 [file DataSheet_1.docx]

Pubmed Database

("tirzepatide" [Supplementary Concept] OR " tirzepatide"[ All Fields] OR " ly3298176"[ All Fields]) AND ("Body Weight"[Mesh] OR "Body Weight Changes"[Mesh] OR "Body Mass Index"[Mesh] OR "Weight Loss"[Mesh] OR "Obesity"[Mesh] OR "Waist Circumference"[Mesh] OR "Adipose Tissue"[Mesh] OR "Body Weight"[ All Fields] OR "Body Mass Index"[ All Fields] OR "Waist Circumference"[ All Fields] OR "Quetelet Index"[ All Fields] OR "BMI"[ All Fields] OR "Weight Reduction"[ All Fields] OR "Weight Losses"[ All Fields] OR "Abdominal Obesity"[ All Fields] OR "Central Obesity"[ All Fields] OR "Visceral Obesity"[ All Fields] OR "obes*"[ All Fields] OR "overweight"[ All Fields] OR "fat mass"[ All Fields] OR "adiposity"[ All Fields] OR "Body Fat"[ All Fields]) AND ("Clinical Trials as Topic"[Mesh] OR "Cross-Over Studies"[Mesh] OR "Double-Blind Method"[Mesh] OR "Single-Blind Method"[Mesh] OR "Random Allocation"[Mesh] OR RCT[All Fields] OR "Clinical Trial" [Publication Type] OR "Controlled Clinical Trials as Topic"[Mesh] OR "Intervention Studies"[ All Fields] OR intervent*[All Fields] OR Trial[All Fields] OR "controlled trial"[ All Fields] OR randomize*[All Fields] OR randomise*[All Fields] OR random*[All Fields] OR placebo[All Fields] OR assignment[All Fields])
