# Supplementary material for: The effects of subcutaneous Tirzepatide on obesity and overweight: a systematic review and meta‐regression analysis of randomized controlled trials
Source: Front Endocrinol (Lausanne). 2023 Aug 9;14:1230206. doi: 10.3389/fendo.2023.1230206 (PMC10446893; doi:10.3389/fendo.2023.1230206)
Supplement: Supplementary file 2 [file Image_1.pdf]

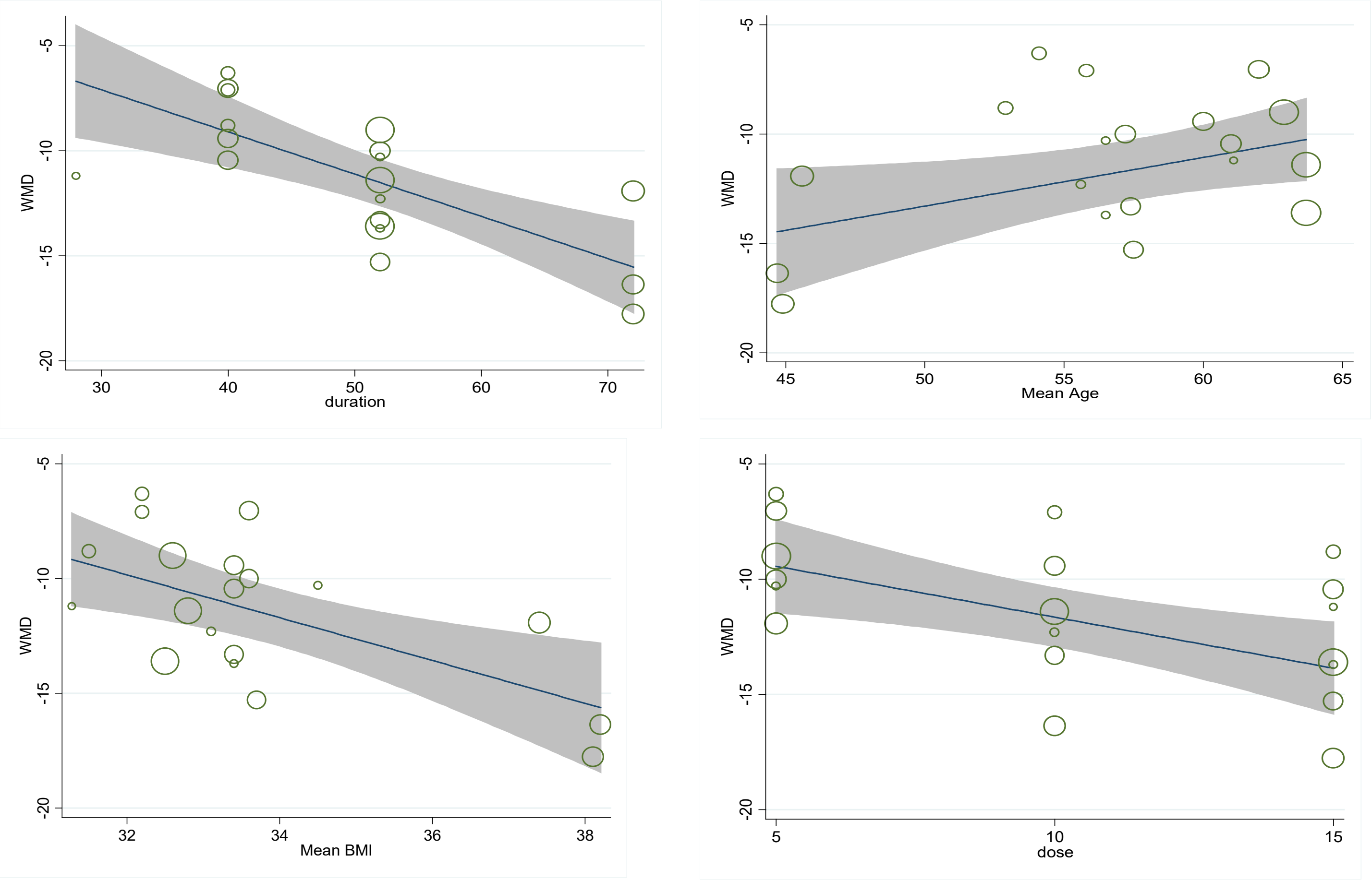

Supplementary Figure 1. Meta-regression analysis encompassing weight changes according to the duration of intervention (weeks), baseline of mean age and BMI, and dose of intervention (mg). *BMI*: Body mass index

A)

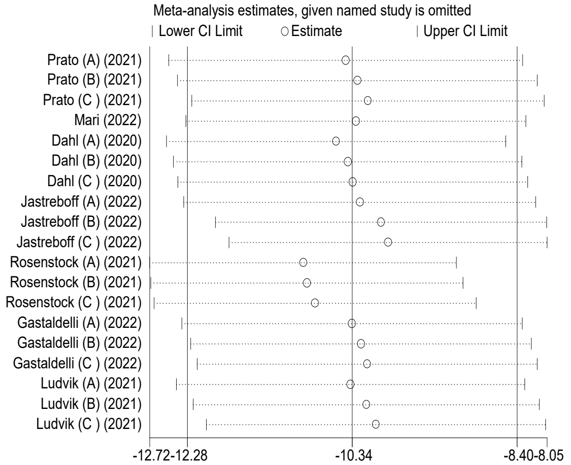

B)

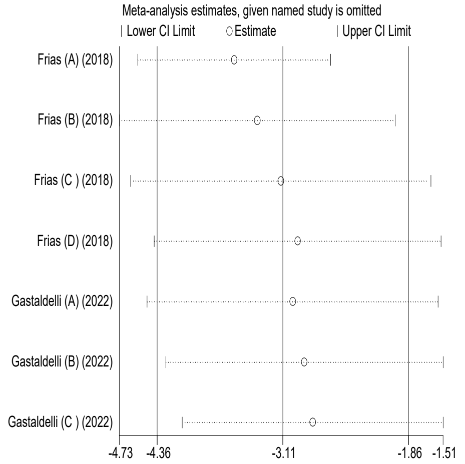

C)

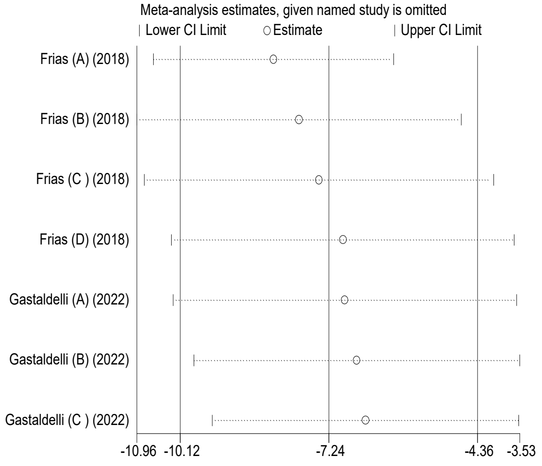

Supplementary Figure 2. Sensitivity analysis of the weighted mean difference (WMD) for A) weight, B) BMI, and C) WC changes. *BMI*: Body mass index, *WC*: Waist circumference

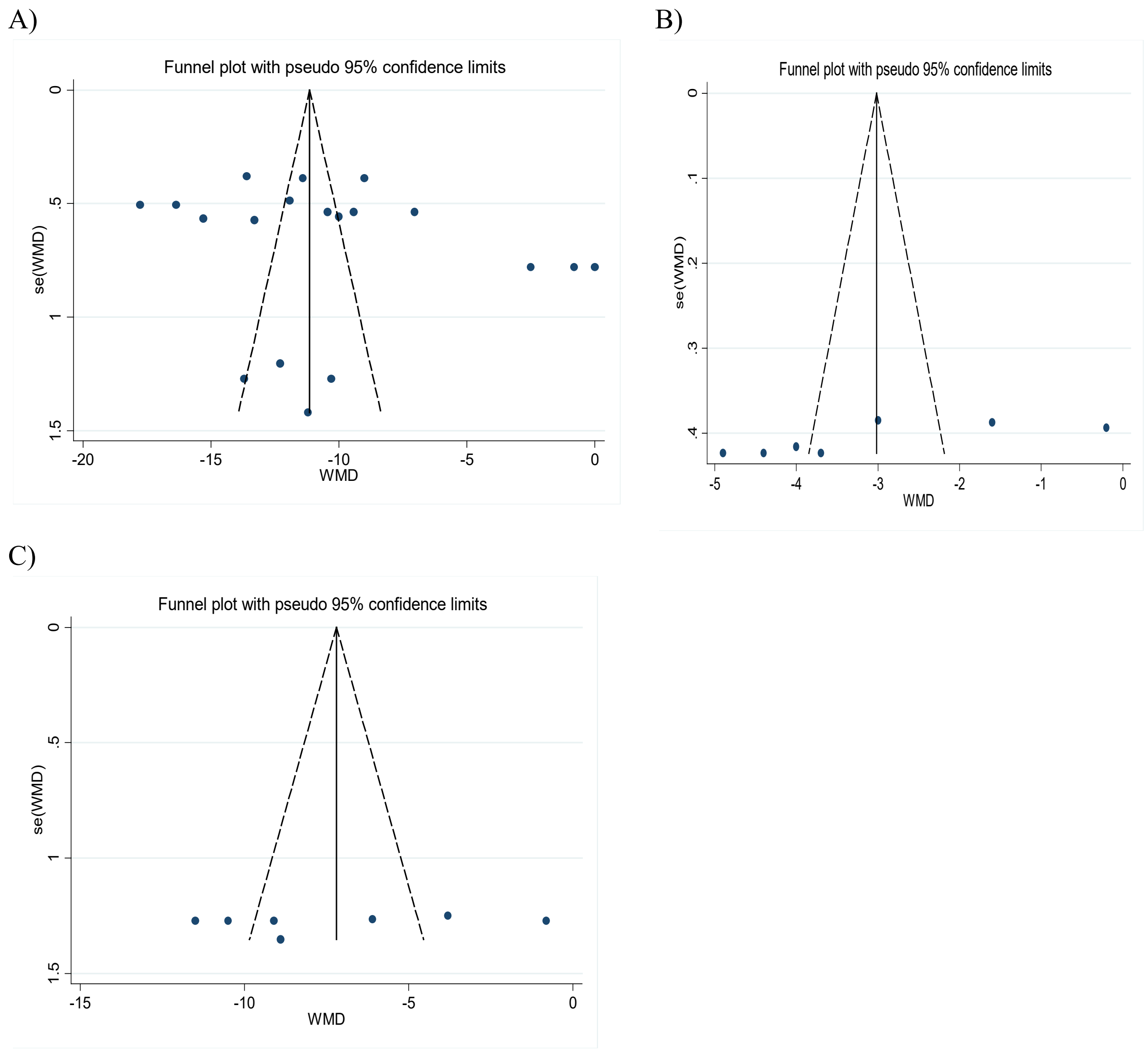

Supplementary Figure 3. Funnel plots for evaluation of publication bias of A) weight, B) BMI, and C) WC. *BMI*: Body mass index, *WC*: Waist circumference
